# One-Step Synthesis of Ag@TiO_2_ Nanoparticles for Enhanced Photocatalytic Performance

**DOI:** 10.3390/nano8121032

**Published:** 2018-12-12

**Authors:** Yufan Zhang, Fan Fu, Yuzhou Li, Desuo Zhang, Yuyue Chen

**Affiliations:** 1 National Engineering Laboratory for Modern Silk, College of Textile and Clothing Engineering, Soochow University, Suzhou 215123, China; yufanzhang21@163.com (Y.Z.); fanfu326@163.com (F.F.); 20165215052@stu.suda.edu.cn (Y.L.); 2 Nantong Textile & Silk Industrial Technology Research Institute, Nantong 226314, China

**Keywords:** Ag@TiO_2_ NPs composite, one-step synthesis, photocatalytic, MO removal

## Abstract

Polyamide network polymers (PNP) modified TiO_2_ nanoparticles (NPs) were decorated with Ag NPs in hydrothermal gel method, forming one-step synthesized photocatalysts, Ag@TiO_2_ NPs. The effect of PNP and the amount of Ag NPs added were investigated in this work. PNP acted as a nanocage to prevent TiO_2_ aggregation and capture Ag accurately, which could effectively control product sizes and improve dispersibility in solvents. Simultaneously, TiO_2_ NPs modified with Ag NPs exhibited remarkable photocatalytic effects. One-step synthesis simplified the experimental process and avoided the agglomeration of silver ions during the secondary reaction, achieving the purpose of uniform distribution at a specific location of TiO_2_ NPs. The prepared Ag@TiO_2_ NPs-0.5 could remove 79.49% of Methyl Orange (MO) after 3 h of ultraviolet light irradiation, which was 2.7 times higher than the reaction rate of pure TiO_2_ NPs. It also exhibited good photoactivity under Visible light conditions. Moreover, the mineralization rate of MO over the Ag@TiO2 NPs-0.5 could be up to 72.32% under UV light and 47.08% under Visible light irradiation, which revealed that the prepared catalysts could effectively degrade most of the MO to CO_2_ and H_2_O. The samples also demonstrated the excellent stability and easy recyclability with over 90% of the original catalytic level for MO degradation. The photocatalysts studied also exerted broad application prospects such as photovoltaic hydrogen production, electronic sensors and biomedicine.

## 1. Introduction

With the rapid advancement of industrialization, human survival faces serious challenges of environmental pollution and energy shortage, which has caused harm to human health [1,2]. Compared with other industries, the textile and dye industry produce 70% of all world annual wastewaters, hence eliminating the dyes in wastewater is especially vital for environmental protection [3,4]. However, till now, traditional methods implemented previously have not been able to remove these organic pollutants from the roots, while semiconductor photocatalytic technology is a viable option to solve this problem [5]. Photocatalytic activity and photostability are important indicators for evaluating photocatalytic quality, but few catalysts have both high photocatalytic activity and excellent light stability, such as oxide semiconductor TiO_2_.

Nanosized TiO_2_, first reported by Fujishima, has caused much attention thanks to its low price, non-toxicity, excellent chemical stability, and wide availability [6,7,8]. However, anatase TiO_2_ can only absorb the wavelengths of sunlight (λ < 400 nm) owing to the wide bandgap (3.2 eV), which greatly limits the utilization of solar energy. Moreover, photogenerated electrons and holes are easily re-combined, resulting in low photocatalytic activity [9,10,11]. Narrowing the band gap of TiO_2_ to broaden the absorption spectrum response interval and reducing the recombination rate of photoinduced electron-hole pairs, have become research hotspots to improve photocatalytic performance [12,13].

In recent years, in order to improve the catalytic effect of TiO_2_ nanoparticles (NPs), surface modification and ion doping were further studied [14,15,16]. The introduction of noble metal to a semiconductor catalyst is one of the effective methods resulting from the synergistic effect of Schottky barrier and surface plasmon resonance [17,18]. On the one hand, the Schottky barrier is formed to accelerate electron/hole separation. On the other hand, TiO_2_ NPs can absorb more photons because of the plasmon resonance effect [19,20]. With its low cost, low toxicity and simple preparation, Ag has become a suitable candidate.

There have been many studies on the modification of TiO_2_ NPs with Ag, while most of them are synthesized by two-step methods, where Ag NPs first are prepared and then combined with TiO_2_ NPs. Oxidation of metal particles, agglomeration of the secondary reaction, and the length of the experiment greatly limit the quality of the product, resulting in insufficient photocatalytic ability of the obtained photocatalyst. In this paper, a one-step synthesis of Ag@TiO_2_ nanoparticles using nanocage as a junction is designed to generate Ag@TiO_2_ in situ. PNP is a polyamide network polymer containing a large amount of amine groups, which is used to surface-modify, as a nanocage, to prevent TiO_2_ NPs aggregation and capture Ag at a fixed position of TiO_2_ NPs [21,22].

To the end, the optimal addition amount of PNP and Ag NPs was obtained by analyzing the origin of the enhancement mechanism and testing the different parameters. The as-prepared composite sample displayed an excellent performance in the catalytic degradation test, because the synergies of Ag and TiO_2_ nanoparticles accelerated the transmission of photogenerated electrons, ultimately increasing the photocatalytic rate, and the results are discussed next.

## 2. Materials and Methods

### 2.1. Materials

Tetrabutyl titanate (TBT), glacial acetic acid (CH_3_COOH, >99%), silver nitrate (AgNO_3_), absolute ethanol (CH_3_CH_2_OH, >99%) were provided by Sigma-Aldrich, St. Louis, MO, USA. Methyl Orange (MO) was supplied from Adamas Reagent Co. Ltd., Shanghai, China. The reagents in this research were analytical grade and required no further purification. Polyamide network polymers (PNP) was formulated to 100 g/L for the following experiment, which were prepared in our previous paper [21].

### 2.2. Preparation of Photocatalysts

#### 2.2.1. Preparation of Pure TiO_2_ Nanoparticles

A total of 10 g TBT was dissolved in 30 mL absolute ethanol, and constantly mixed with ultrasonic for 0.5 h. The obtained mixed solution is referred to as liquid A. Moreover, 60 mL of Liquid B was placed in a three-necked flask containing deionized water, glacial acetic acid, and absolute ethanol at a volume ratio of 1:1:4. Then, liquid A was dripped into Liquid B at a rate of 1 mL per minute under stirring for up to 2 h, at ambient temperature. The gel was formed after 24–36 h, with the color changing from transparent to light blue.

A total of 1 mol PNP solution (100 g/L) was added into 10 g gel and kept under magnetic stirring until completely dissolved. Afterwards, the mixture was poured into a 100 mL polytetrafluoroethylene lining, which was kept at 200 °C for 8 h in an autoclave. After naturally cooling to room temperature, the obtained product was centrifuged multiple times to eliminate excess precursor and PNP until the pH of the solution returned to neutral. Finally, the TiO_2_ NPs white in color were obtained under 60 °C dry conditions.

#### 2.2.2. Preparation of Ag@ TiO_2_ Nanoparticles

In this study, Ag@TiO_2_ NPs were one-step produced with AgNO_3_ as metal source and TBT as the precursor. According to this procedure, 10 g TBT was dispersed in absolute ethanol at a volume ratio of 1:3. The above solution was then added dropwise into a 60 mL mixture solution, which was made up of 10 mL deionized water, 40 mL absolute ethanol and 10 mL glacial acetic acid. After stirring for 3 h at 20 °C, the solution turned into gels. A suspension composed of 10 g gels, 1 mol PNP solution (100 g/L) and 0.1 M AgNO_3_ solution was stirred for 2 h. Next, the mixture was poured into a 100 mL polytetrafluoroethylene lining and hydrothermally treated for 8 h at 200 °C. In order to optimize the molar ratio of Ag to TiO_2_, Ag@TiO_2_ with different molar ratios of AgNO_3_ (0.25%, 0.5% and 1%) were prepared and the resulting samples were labelled as Ag@TiO_2_ NPs-x, where x was 0.25, 0.5, and 1, respectively. In all cases, the samples were washed thoroughly to get rid of excess precursor and PNP solution and then stored at 60 °C in a vacuum oven until required.

### 2.3. Materials Characterization

The Hitachi S-4800 scanning electron microscopy (Hitachi Ltd. Tokyo, Japan) was used to show the images of the samples surface morphology and particle size distribution. In order to better analyze the morphology and particle size, transmission electron microscopy with EDS capabilities (TEM), high-resolution electron microscopy (HRTEM) and selected area electron diffraction (SAED) were tested on a Tecnai G2 F20 S-TWIN (FEI, Hillsboro, OR, USA) operating at 200 kV. The X-ray diffraction scanning rate of 5° min^−1^ characterized the crystal form of the products, with Cu-Kα (λ = 0.1542 nm), equal voltage of 40 KV and equal current of 200 mA. All data from 20° to 80° in the XRD spectrum were integrated and recorded, and the characteristic peaks were determined by comparison with the Joint Committee on Powder Diffraction Standards (JCPDS 21-1272 and JCPDSCard No. 04-0783) [23,24]. PHI-5700 ESCA apparatus was operated to test the X-ray photoelectron spectroscopy. It could be seen from the spectrum that all the values of binding energy were adjusted with the help of the hydrocarbon band (C 1 s: 284.8 eV), the X-ray source expressed Al Ka (hv = 1486.6 eV). Fourier transform infrared spectra (FTIR) of pure TiO_2_ NPs and Ag@TiO_2_ hybrid NPs were measured on a Nicolet 5700 instrument (Thermo Electron Corporation, Beverly, MA, USA). Evolution 220 UV-Vis spectrophotometer (Thermo Fisher Scientific, Boston, MA, USA) was used to detect the absorption capacity of the prepared hybrid catalysts. It should be noted that integrating sphere method using BaSO_4_ powder as the background was carried out in this experiment, where the measurement range was 200–800 nm. Simultaneously, the total MO contents in the tests were also recorded (λ = 463 nm).

### 2.4. Photoelectrochemical Measurements

The photoelectrical response and electrochemical impedancespectroscopy (EIS) play vital roles in evaluating the separation efficiency of the photogenerated carriers. The photoelectrochemical measurements were performed in a standard three electrodes system with a 0.1 M Na_2_SO_4_ aqueous electrolyte solution using a PGSTAT302N electrochemical workstation (Swiss Wantong China Ltd., Shanghai, China). Platinum wire and saturated calomel electrode would serve as the counter electrode and the reference electrode, respectively. The working electrode was obtained by dipcoating 3 μm of the suspension onto glassy carbon electrode. Photo-responses were measured at 0.0 V and the EIS measurements were defined at a 5 mV AC voltage amplitude with a frequency range of 10^5^ to 10^−1^ Hz. The working electrodes were irradiated by a 300 W Xe lamp. The set light-liquid interface distance was 15 cm, the light intensity was 50 mW cm^−2^ and the d of the glassy carbon electrode was 3 mm.

### 2.5. Photocatalytic Activity Measurements

The ability of photodegradation of dye determined the photocatalytic performance of the catalyst, and the as-prepared catalyst was placed into MO for photodegradation reaction to evaluate the effect. A 20 W UV light lamp (365 nm) and 300 W Xe lamp (equipped with a 420 nm filter) were applied as the light source to induce photocatalysis, and light intensity for both was 50 mW cm^−2^. The set light-liquid interface distance was 15 cm. According to previous standards, 100 mg catalysts were dispersed in beaker containing 100 mL MO (20 mg/L) and the solution was thoroughly mixed for approximately 15 min. Considering the adsorption-desorption between the sample and the dye, the test solution needed to be pretreated in the dark prior to the experiment [25]. Afterwards, the suspension was stirred for 3 h under irradiation and 3 mL of the droplet was uniformly sampled at a given time interval into a tube, centrifuging for testing.

By measuring the maximum absorption of the MO characteristic peak at 463 nm, the absorption standard curve equations for MO could be depicted on a UV-Vis spectrophotometer. The mineralization rates of MO were obtained using the total organic carbon (TOC) equipment (Multi N/C 2100, TOC/TN, Analytikjena, Jena, Germany). Reusability was another key indicator for determining the performance of the catalyst, hence 100 mg of Ag@TiO_2_ NPs was subjected to cyclic photocatalytic testing five times in a row. During the test, the MO concentration was always set to 20 mg/L. After each cycle, the catalyst was collected by centrifugation and dried at 60 °C for the next usage.

## 3. Results and Discussion

### 3.1. Preparation of Ag@TiO_2_ NPs

**Figure 1:**
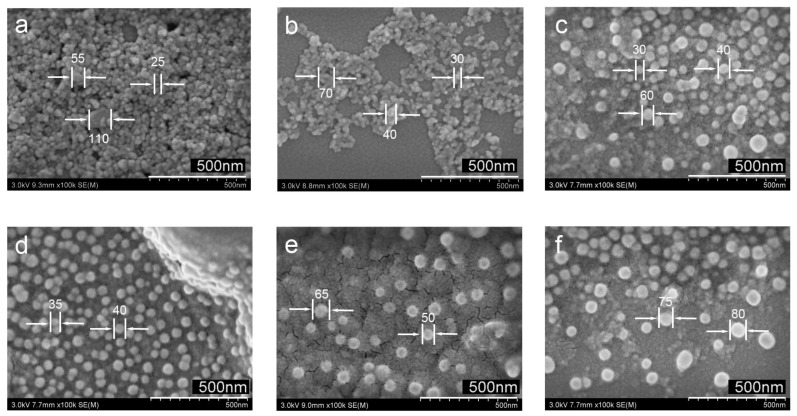
SEM images of TiO_2_ nanoparticles (NPs) with different molars of polyamide network polymers (PNP): 0 mol (**a**), 0.25 mol (**b**), 0.5 mol (**c**), 1 mol (**d**), 2 mol (**e**) and 3.125 mol (**f**), respectively.

For nanocatalysts, particle size and dispersibility in solvents are two important parameters. Surfactants or polymers are typically added during the preparation to control the size of the nanoparticles and increase the dispersibility in the solvent. In this work, polymer PNP was used as a template to find the appropriate amount added without metal loading. In order to clearly verify the change in diameter and dispersion of TiO_2_ nanoparticles, SEM was employed for further examination. Figure 1 depicts the SEM images of the TiO_2_ NPs synthesized by the PNP in the conditions of different molars. As shown in Figure 1a, the pure TiO_2_ NPs have a small particle size but have serious agglomeration owing to the high surface energy, which results in poor dispersibility as well as extremely large size ranges. After that, by adjusting the amount of PNP added to 0.25, 0.5 and 1 mol (Figure 1b–d), respectively, more and more PNP is added for the preparation of the TiO_2_ NPs, hence the growing nanoparticles are confined by the polymeric nanocages in order to prevent from aggregating [26,27]. Compared with the pure TiO_2_ NPs, the agglomeration phenomenon and the dispersibility are gradually improved. It follows that the overall size is controlled in the range of 30–40 nm after adding 1 mol of PNP, and most of the synthesized nanoparticles show good dimensional uniformity, which indicates a suitable amount of PNP solution can effectively improve the dispersion ability and keep the particle size at a small level. However, as the amount of PNP added exceeds 1 mol (Figure 1e,f), the mean diameter would become larger, which is caused by the steric hindrance generated with a large number of cavities between the branches. Thus, 1 mol PNP (100 g/L) was selected to prepare Ag@TiO_2_ NPs for the following work.

**Scheme 1:**
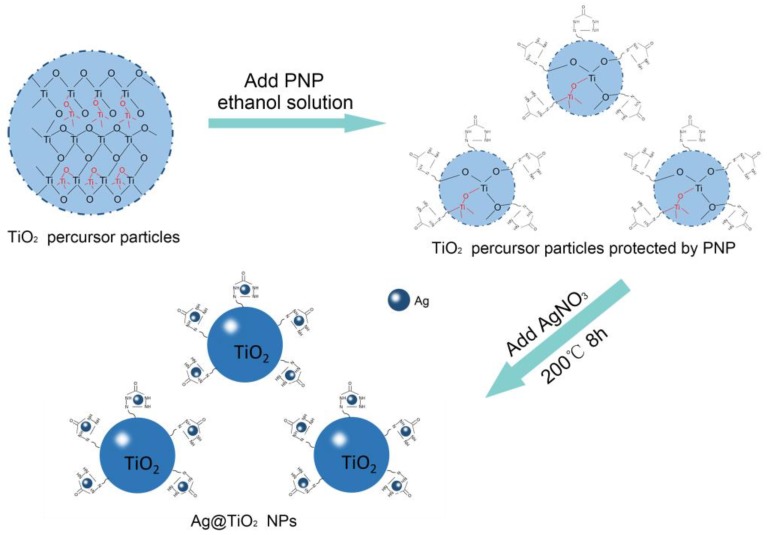
Procedure for the synthesis of Ag@TiO_2_ NPs.

The key steps of designing the pure TiO_2_ NPs and Ag@TiO_2_ NPs by the hydrothermal gel method are illustrated in Scheme 1. The first step of our approach was to prepare the TiO_2_ precursor obtained by hydrolyzing TBT, as Ti source, into a gel form. To prevent the aggregation in the next hydrothermal reaction, prepared PNP was introduced for protecting TiO_2_. Finally, Ag@TiO_2_ NPs was synthesized under high temperature and high pressure conditions, and the color turned a deep reddish brown.

Our previous works have found that by using PNP as a nanocage, a polyamide network polymer that contains numerous amine groups, metal particles can be complexed with amine groups and reduced to Ag NPs at a fixed position in the network structure during the initial stage of hydrothermal reaction [28]. Since the space of the network structure is limited, the size of the Ag NPs grown inside will be restricted, so that products with small size and uniform distribution are obtained. Meanwhile, the surface of the Ag NPs is rich in amino groups, which can be linked to the hydroxyl groups on the surface of the TiO_2_ by hydrogen bonding, thereby achieving the effect of physically preventing aggregation [22]. These two findings inspired us to design a method to complete the growth of Ag and TiO_2_ NPs in one-step.

**Figure 2:**
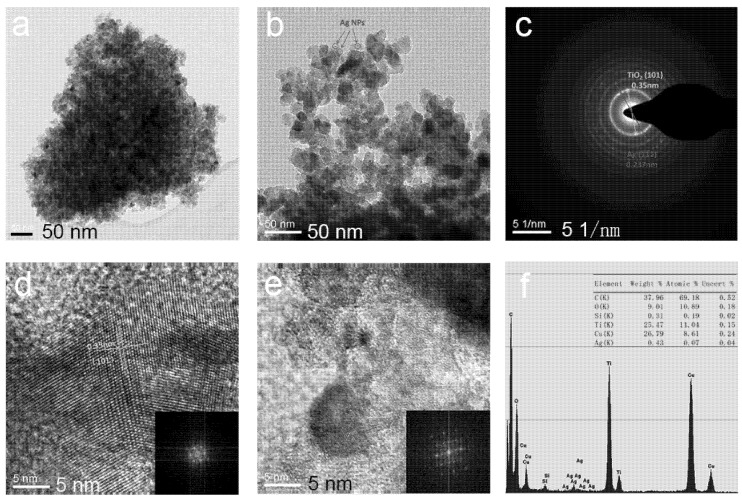
Transmission electron microscopy with EDS capabilities (TEM) image of the pure TiO_2_ NPs (**a**) and Ag@TiO_2_ NPs-0.5 (**b**); selected area electron diffraction (SAED) image of Ag@TiO_2_ NPs-0.5 (**c**); high-resolution electron microscopy (HRTEM) image taken from TiO_2_ NPs (**d**) and the corresponding fast Fourier transform (FFT) mode; HRTEM image taken from Ag NPs (**e**) and the corresponding FFT mode; EDS of Ag@TiO_2_ NPs-0.5 (**f**).

After loading the silver particles, the morphology, structure and property of the photocatalyst were also changed. To further explore the morphologies, the lattice parameters and the element content of the samples, TEM, SAED, HRTEM and EDS were employed. The results are displayed in Figure 2 below. It is depicted that pure TiO_2_ NPs (Figure 2a) have undergone severe agglomeration, whereas Ag@TiO_2_ NPs-0.5 (Figure 2b) show excellent dispersibility and small particle size under the synergy of PNP and Ag NPs. It can clearly see that Ag NPs (d <10 nm) are embedded on the surface of TiO_2_ NPs from the low magnification TEM image, as shown in Figure 2b, which also proves the existence of Ag NPs. A SAED pattern of Ag@TiO_2_ NPs-0.5 is indicated in Figure 2c. The (111) crystal plane of Ag NPs and the (101) crystal plane of anatase are confirmed to match the d values obtained in the results. Meanwhile, the HRTEM image of TiO_2_ and Ag (d is around 8 nm), accompanied by a fast Fourier transform (FFT) mode of local regions (inset), which exhibits the lattice fringes of TiO_2_ (101) and Ag (111) planes with interplanar spacing of about 0.35 and 0.237 nm, respectively, are observed in Figure 2d,e [29]. The results are consistent with the results obtained from the diffraction pattern, indicating the as-prepared Ag@TiO_2_ NPs is high quality. Figure 2f depicts the EDS of Ag@TiO_2_ NPs-0.5, and the content of each element is also listed in the table where the Ag atom content is small, accounting for 0.6% of the Ti atom, which is also consistent with the experimentally set ratio.

**Figure 3:**
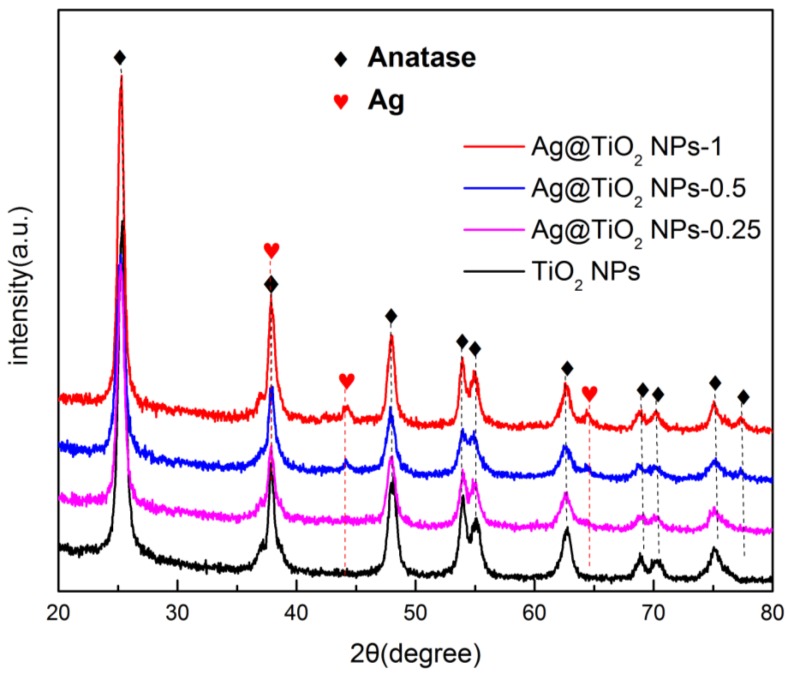
X-ray diffraction (XRD) patterns of pure TiO_2_ NPs, Ag@TiO_2_ NPs with different molar ratios of 0.25%, 0.5% and 1%.

XRD patterns were utilized to characterize the structure of as-prepared catalyst. Figure 3 shows the test results of TiO_2_ NPs and Ag@TiO_2_ NPs prepared through the one step in situ hydrothermal method. The Ag@TiO_2_ NPs are mainly composed of anatase and nano silver, which are marked “Anatase” and “Ag” in the image. It is shown that the diffraction peaks at Bragg angles (2θ) of 25.3, 37.9, 48.05, 53.9, 55.06, 62.4, 68.76, 70.3, 75.06, 78.67 correspond to the (101), (004), (200), (105), (211), (204), (116), (220), (215), (206) lattice planes of anatase TiO_2_ (JCPDS 21-1272). As observed, the crystal shape of the diffraction peaks appear clear and sharp, and there are no other impurity peaks, indicating well-formed crystal structures of the as-prepared TiO_2_. In addition, the (200), (220) lattice planes at Bragg angles (2θ) of 44.2 and 64.4 (JCPDSCard No. 04-0783) belong to Ag consistent with the TEM results, the peak intensities of which become stronger with an increase in the molar mass. It is noteworthy that since the diffraction band of the Ti (004) lattice plane is too high at 37.9°, the (111) characteristic peak of Ag is covered. Using the formula Scherrer Equation (1), the particle size corresponding to each crystal plane can be theoretically calculated [30,31]. Taking Ag@TiO_2_ NPs-0.5 as an example, the mean diameter of TiO_2_ NPs is estimated at 17, nm consistent with TEM results.

(1) $$D=\frac{K\lambda }{\beta \cos\theta },$$

where K expresses a constant with a value of 0.89, the X-ray incident wavelength is represented by λ with a value of 0.154 nm, θ represents the half-diffraction angle and β is the full width at half maximum (rad).

**Figure 4:**
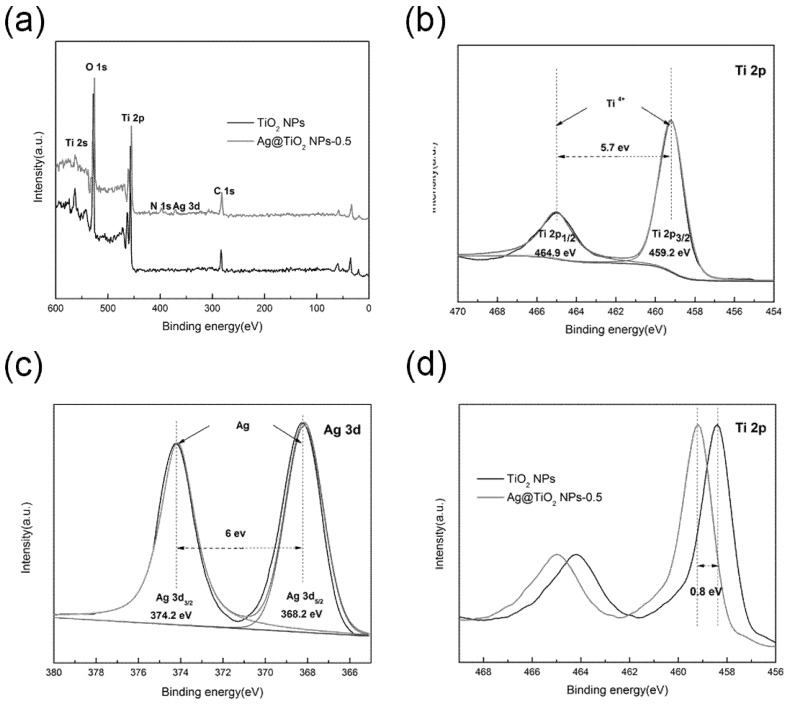
XPS spectrum of TiO_2_ NPs and Ag@TiO_2_ NPs-0.5 (**a**); high resolution spectra of Ti 2p (**b**); Ag 3d (**c**); Ti 2p of TiO_2_ NPs and Ag@TiO_2_ NPs-0.5 (**d**).

XPS was implemented to further investigate the valence state of Ti and Ag atoms in Ag@TiO_2_ NPs. The C, O, N, Ti and Ag elements can be observed in the survey spectrum (Figure 4a), whereas the C1s at 286.8 eV is used as a standard to determine the characterization data. Taking Ag@TiO_2_ NPs-0.5 as an example, the XPS narrow-spectrum Ti 2p is shown in Figure 4b, and the binding energy generated by the spin-orbital splitting of the Ti 2p_1/2_ and Ti 2p_3/2_ peaks are 464.9 and 459.2 eV, which clearly proves that Ti exists in the form of Ti^4+^ in the sample [32,33,34]. Furthermore, compared with pure TiO_2_ NPs, the binding energy at Ti 2p (Figure 4d) increases by approximately 0.8 eV as the amount of AgNO_3_ is added. This phenomenon is caused by the difference in Fermi level between TiO_2_ and Ag, where electrons are transferred from TiO_2_ with high energy level to Ag until the energy levels are equal, resulting in a decline in the density of external electron clouds for Ti ions, and an increase in binding energy [35]. Figure 4c exhibits two characteristic peaks of the Ag 3d spectrum, where the binding energy of Ag 3d_3/2_ is 374.2 eV and Ag 3d_5/2_ is 368.2 eV. Carefully compared with Ag@TiO_2_ NPs-1 and Ag@TiO_2_ NPs-0.25 (Figure S1a,b), it can be seen that the splitting width between Ag 3d_3/2_ and Ag 3d_5/2_ are also 6 eV, indicating the presence of Ag^0^ chemical states in the Ag@TiO_2_ NPs [36].

**Figure 5:**
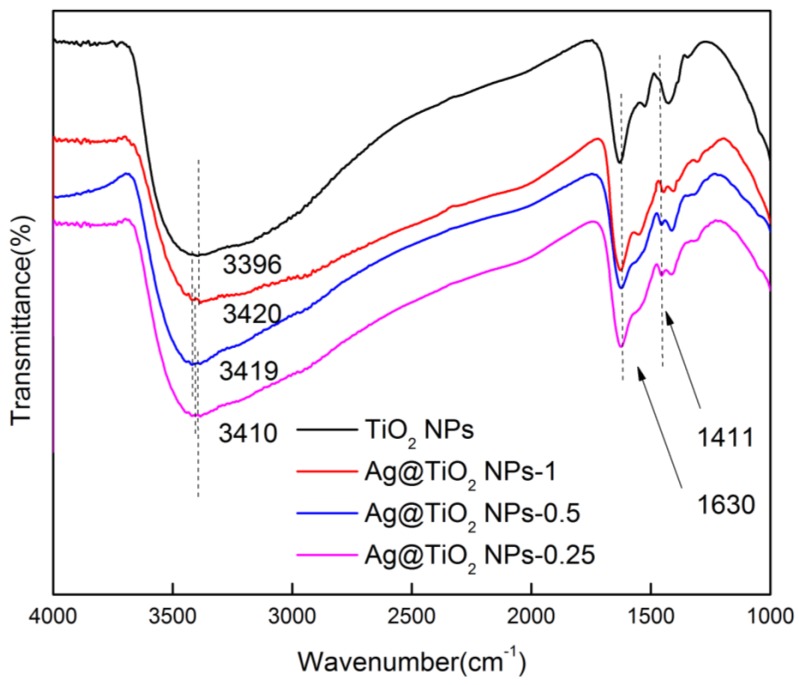
Fourier transform infrared spectra (FTIR) spectra of pure TiO_2_ NPs and Ag@TiO_2_ NPs.

Figure 5 shows the FTIR spectrum of TiO_2_ NPs and Ag@TiO_2_ NPs catalysts. It shows that the strong absorption peak of TiO_2_ NPs and Ag@TiO_2_ NPs at 3396, 3410, 3419 and 3420 cm^−1^ have similar behavior, which is likely due to hydrogen bonded surface hydroxyl groups and OH of adsorbed water molecules [37,38,39,40]. In addition, the bending vibration peak of the O–H bond can be clearly observed around 1630 cm^−1^. The signal at 1411 cm^−1^, a narrow peak of the alcohol O–H bond bending vibration also appears, which is due to the addition of PNP ethanol solution [41].

### 3.2. Photocatalytic Activity of Ag@TiO_2_ NPs

Photocatalytic ability is a vital factor in evaluating photocatalysts. Figure S2 shows the UV–Vis diffuse reflectance spectroscopy. The initial absorption edge of pure TiO_2_ NPs is estimated to be 380 nm with a strong light absorption in the ultraviolet area. The modifications of the Ag NPs can enhance the photocatalytic activity under ultraviolet light, as well as Visible light. With regard to the Ag@TiO_2_ samples with different loading amounts, compared to that of pure TiO_2_ NPs, it can be observed that the absorption edges show a slight red shift which also enhances its Visible light photocatalytic activity to some extent [42,43,44,45]. Particularly, Ag@TiO_2_ NPs-0.5 has the strongest light absorption among these catalysts. This is mainly owing to the suitable content deposition of Ag NPs, which can effectively respond to Visible light caused by plasmon resonance effect and suppress the recombination of electron/hole pairs in order to promote the transfer and transport of photogenerated carriers. However, the excessive number of Ag nanoparticles leads to aggregating together, inhibiting the absorption of light, and providing an active site for the recombination of electron/hole pairs, thus, the light absorption ability is lowered.

**Figure 6:**
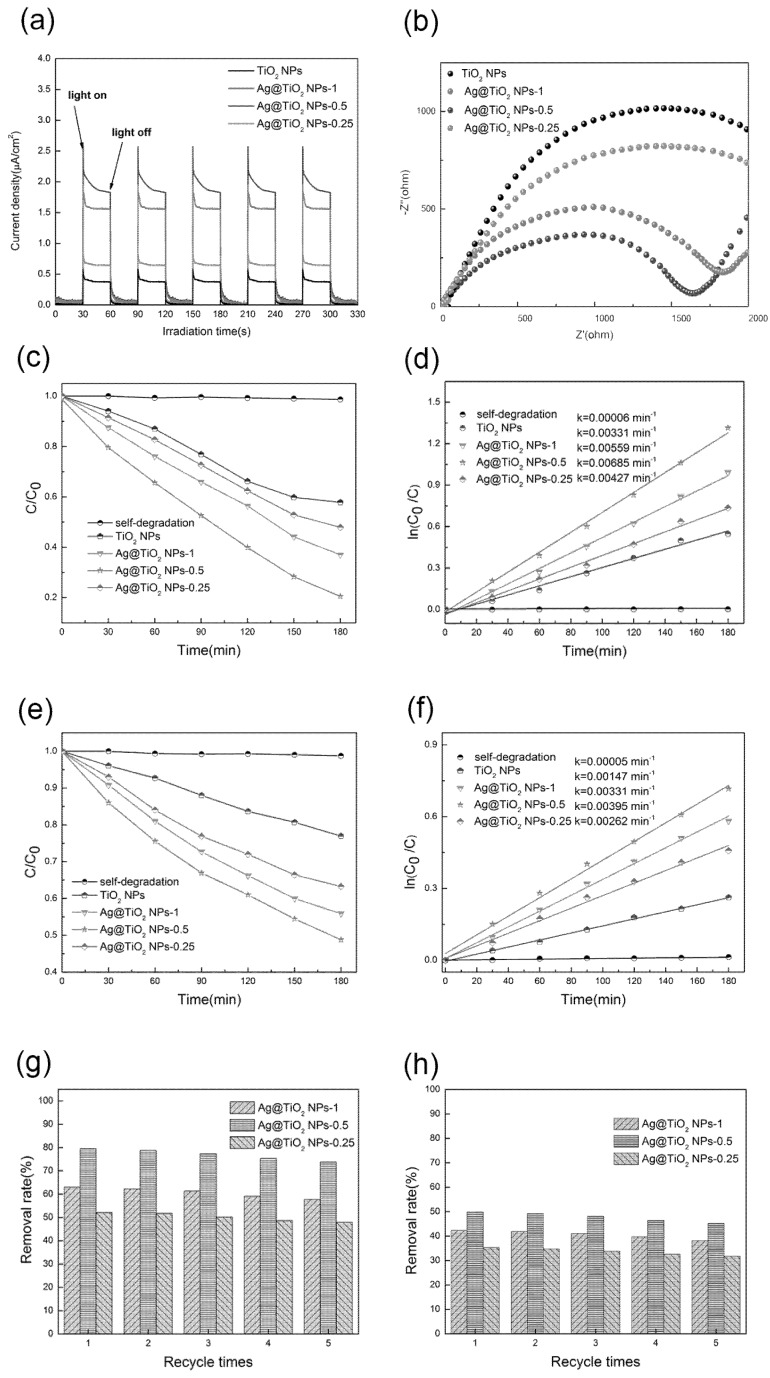
Photocurrent responses spectroscopy (**a**) and electrochemical impedance spectroscopy (EIS) (**b**) of the TiO_2_ NPs and Ag@TiO_2_ NPs samples; Methyl Orange (MO) photocatalytic degradation curves of the TiO_2_ NPs and Ag@TiO_2_ NPs samples under UV light (**c**); MO photocatalytic degradation Kinetic fit of the TiO_2_ NPs and Ag@TiO_2_ NPs samples under UV light (**d**); MO photocatalytic degradation curves of the TiO_2_ NPs and Ag@TiO_2_ NPs samples under Visible light (**e**); MO photocatalytic degradation Kinetic fit of the TiO_2_ NPs and Ag@TiO_2_ NPs samples under Visible light (**f**); Cycling performance of Ag@TiO_2_ NPs samples for MO degradation under UV light (**g**); Cycling performance of Ag@TiO_2_ NPs samples for MO degradation under Visible light (**h**).

Photocurrent measurement was carried out to evaluate the electron–hole recombination in the as-prepared samples (Figure 6a), where the photocurrent intensity remained steady and reproducible over several intermittent on-off cycle experiments. Apparently, the Ag@TiO_2_ NPs-0.5 have the strongest photocurrent response which is ~4 times higher than that of pure TiO_2_ NPs, suggesting that the Ag@TiO_2_ NPs-0.5 have more effective charges separation and thus inhibit the recombination of electrons and holes under Visible light irradiation [46,47]. Moreover, electrochemical impedance spectra (EIS) measurements were performed to elucidate the charges transfer process of the photogenerated charge carriers. It is generally known that the radius of the Nyquist circle is directly related to charge transfer resistance, with a smaller semicircle radius corresponding to easier electron transfer. From Figure 6b, the radius of the arc in the EIS spectrum of Ag@TiO_2_ NPs-0.5 is smaller than that of TiO_2_ NPs and Ag@TiO_2_ NPs, indicating that highly efficient interfacial charges transfers occur in Ag@TiO_2_ NPs-0.5.

The photocatalytic abilities of Ag@TiO_2_ NPs samples were assessed through the degradation of MO. To rule out the interference of MO self-degradation, we performed a self-degradation test on MO. In the condition of no photocatalyst and irradiation for 180 min, nearly no degradation of MO was found, which indicates that MO dye is suitable as a targeted pollutant and available to assess the catalytic ability of the catalyst owing to its steady structure. Figure 6c shows the photodegradation efficiencies after the UV light irradiating for 3 h. The photodegradation efficiency decreases in the order below:

Ag@TiO_2_ NPs-0.5 > Ag@TiO_2_ NPs-1 > Ag@TiO_2_ NPs-0.25 > TiO_2_ NPs.

The MO photodegradation efficiencies are estimated to be 79.49%, 62.98%, 52.05% and 42.14%, respectively. Clearly, as the molar ratio of Ag increases in a certain range, the degradation of the sample also increases, which implies that Ag plays an important role in photocatalytic degradation. Studies have shown that the photodegradation process of MO conforms to the model of Langmuir–Hinshelwood first-order reaction kinetics. Therefore, the reaction rate constant could be obtained by calculation through the following equation:

ln(C_0_/C) = k × t, (2)

in which k and t express the rate constant (min^−1^) of photocatalytic degradation of MO and the irradiation time (min), respectively, while C_0_ as well as C represent the concentration of MO after adsorption equilibrium and reaction time, respectively. Figure 6d proposes the linear fit of the photocatalytic degradation time against ln(C_0_/C). It is worth noting that the catalytic reaction rate of Ag@TiO_2_ NPs-0.5 is 2.7 times higher than TiO_2_ NPs, which exhibits the optimum catalytic performance among photocatalysts above [48].

The photodegradation efficiencies under Visible light irradiation for 3 h are shown in Figure 6e, which has similar behavior to the catalysis under UV conditions. Ag@TiO_2_ NPs-0.5 shows the strongest photocatalytic effect, and 50% of MO is decomposed after 180 min irradiation. To better compare the photocatalytic performance of these samples, the reaction kinetics of MO degradation is also investigated in Figure 6f. Obviously, Ag@TiO_2_ NPs-0.5 has the maximum k value of 0.00395 min^−1^, which is almost 2.6 times as high as that of TiO_2_ NPs.

In addition, the UV-absorption spectrum of the Ag@TiO_2_ NPs-0.5 suspension was recorded during the process of photocatalytic degradation. As the UV light irradiation time increases, it is apparent that the MO absorption band intensity decreases gradually. As shown in Figure S3a, after 60 min, the band intensity decreases rapidly with time and has been levelled off after 150 min. According to previous experiments, the chromophoric group of MO is the azo group (–N=N–), which corresponds to the wavelength of 463 nm [49]. The absorption maximum of band intensity at 463 nm was measured to estimate the concentration of MO in the solution and the catalytic effect can be visually observed. The corresponding color changes using Ag@TiO_2_ NPs-0.5 are also depicted in the Figure S3b. Moreover, a total organic carbon (TOC) analysis was performed under UV and Visible light condition to further confirm the degree of oxidation (Figure S4). Through the measurement of TOC analyzer, the mineralization rate of MO over the Ag@TiO_2_ NPs-0.5 can be up to 72.32% under UV light and 47.08% under Visible light irradiation. Compared with the photocatalytic degradation curves of TiO_2_ NPs and Ag@TiO_2_ NPs samples (Figure 6c,e), the TOC degradation efficiency of MO is relatively lower than the degradation rates, which proves that MO is decomposed into CO_2_ and H_2_O, accompanied with the formation of a certain intermediates in the process of photocatalytic degradation [50,51].

The stability and repeatability of the catalyst are of decisive significance in practical applications. In order to get insights into the reusability, the recycling capability of the photocatalyst is carried out with the Ag@TiO_2_ NPs samples. In our test, the nanocomposite photocatalysts of MO were collected by centrifugation and then replaced into the same concentration of MO solution after each photodegradation. As displayed in Figure 6g,h, the removal rate after five consecutive runs is only slightly changed and on the 5th regeneration cycle, the degradation efficiency of the MO under UV light is higher than 90% of the initial amount, as well as under the Visible light condition [52].

**Scheme 2:**
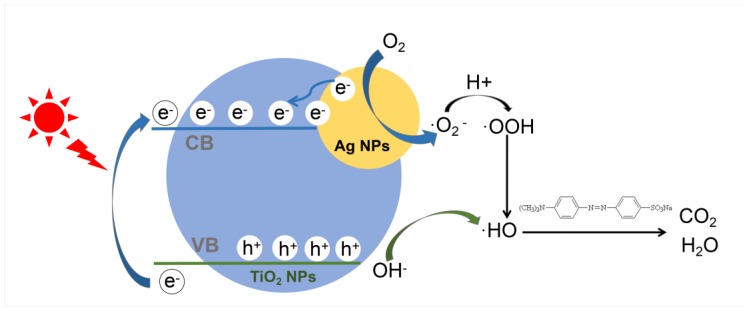
Schematic diagram of electron transfer in Ag@TiO_2_ NPs and degradation mechanism of MO.

It can be seen in Scheme 2 that a credible approach to enhance photocatalytic activity is proposed based on experimental analysis and discussion. On the one hand, Ag deposited NPs, a Schottky barrier has appeared at the interface between metal and semiconductor in UV light as a result of the lower Fermi level of Ag, and then the photoinduced electrons are transferred from the CB of TiO_2_ to the Ag nanoparticles, promoting electron/hole pairs to separate [53,54]. What is more, the lifetimes of the charge carriers are increased, and the photocatalytic activity is improved because of the separation of photogenerated electrons and holes in this process. On the other hand, caused by the surface plasmon resonance effect, Ag nanoparticles are easily excited in the condition of being exposed to Visible light, which produces the large number of electrons on the surface. These electrons are transferred to the CB to enhance the absorbance value of TiO_2_ in the Visible region [55,56]. During the photocatalytic process, the electrons accumulated on the TiO_2_ react with the oxygen molecules existed on the surface or dissolved in the water, and are reduced to the activated superoxide anion radical∙O_2_^−^, which can remove organic pollutants or further generate oxides through a series of reactions with H^+^. Meanwhile, holes can oxidize -OH or H_2_O to strong oxidizing ∙OH radicals. Finally, strong oxidizing radicals ∙OH will oxidize MO to form CO_2_ as well as a small number of partially oxidized intermediates [57,58].

## 4. Conclusions

In this work, a composite photocatalyst of TiO_2_ NPs decorated with Ag NPs was prepared by the method of one-step synthesis, and the mass moral of PNP is also an important part in the dispersion and size of the growing particles in the hydrothermal reaction. The results indicate that Ag@TiO_2_ NPs-0.5 shows the highest level of degradation efficiency, the mineralization rate of MO and shows the significant absorption range of Visible light. Beyond that, the Ag@TiO_2_ NPs exhibit outstanding recyclability and retain over 90% of the original catalytic level for MO after five cycles of recycling. Therefore, there is a broad developing prospect in this work of photocatalytic hydrogen production, solar cells, sensors, rechargeable electrodes and exhaust gas treatment.

## Supplementary Materials

The following are available online at http://www.mdpi.com/2079-4991/8/12/1032/s1; Figure S1: Ag 3d high resolution spectra of Ag@TiO_2_ NPs-1 (a) and Ag@TiO_2_ NPs-0.25 (b); Figure S2. Optical absorption spectroscopy; Figure S3: UV absorption spectrum during MO degrade process under UV light; Figure S4: Mineralization rates of MO by the samples under UV and Visible light irradiation. Samples 1–4 denote pure TiO_2_ NPs, Ag@TiO_2_ NPs-0.5, Ag@TiO_2_ NPs-0.25 and Ag@TiO_2_ NPs-1.
