# Supplementary material for: One-Step Synthesis of Ag@TiO2 Nanoparticles for Enhanced Photocatalytic Performance
Source: Nanomaterials (Basel). 2018 Dec 12;8(12):1032. doi: 10.3390/nano8121032 (PMC6316728; doi:10.3390/nano8121032)
Supplement: Supplementary file 1 [file nanomaterials-08-01032-s001.pdf]

Supplementary Materials

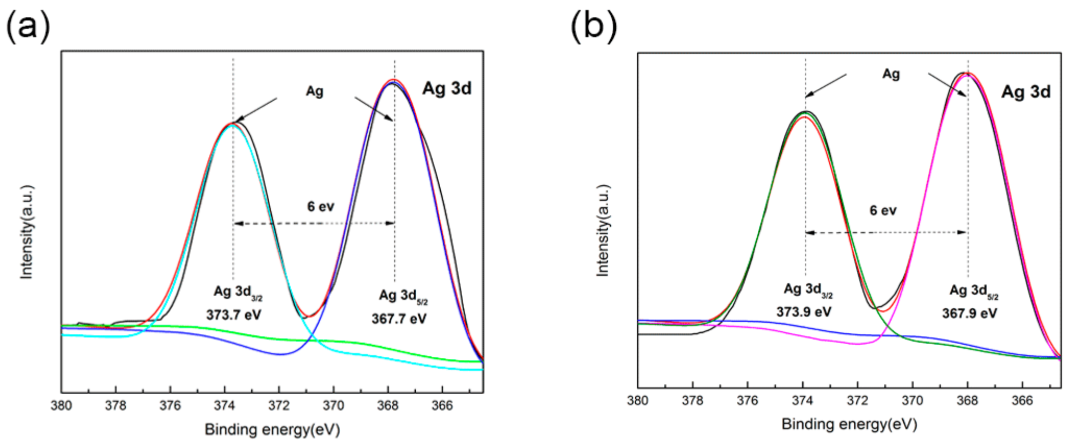

Figure S1. Ag 3d high resolution spectra of Ag@TiO2 NPs-1 (**a**) and Ag@TiO2 NPs-0.25 (**b**).

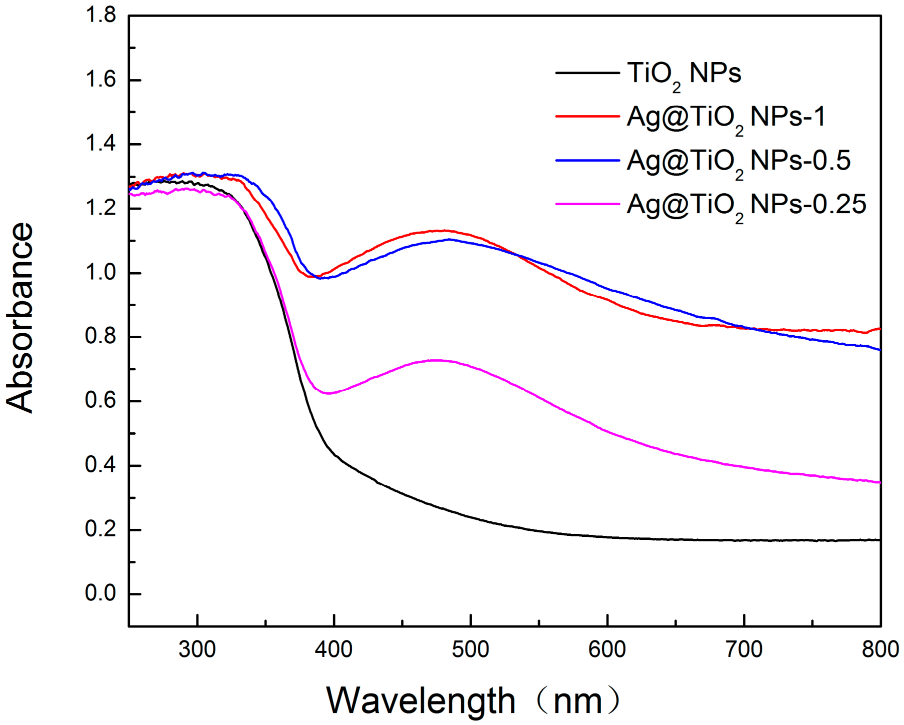

Figure S2. Optical absorption spectroscopy.

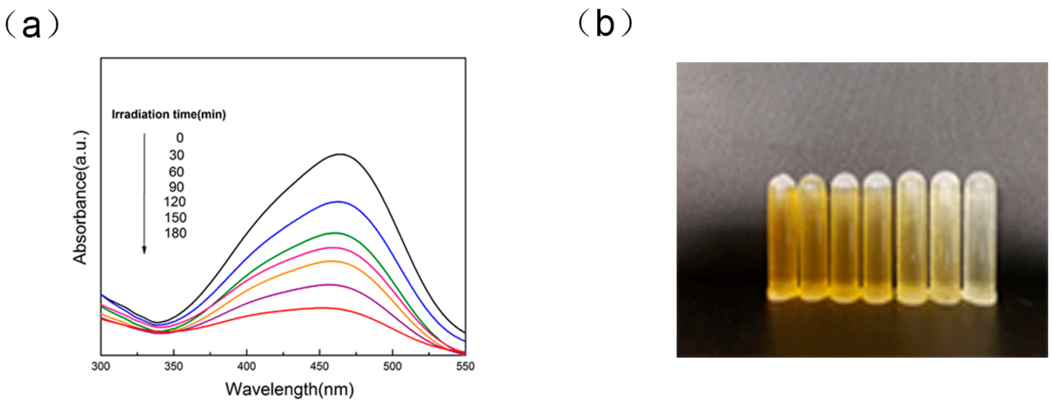

Figure S3. UV absorption spectrum during MO degrade process under UV light.

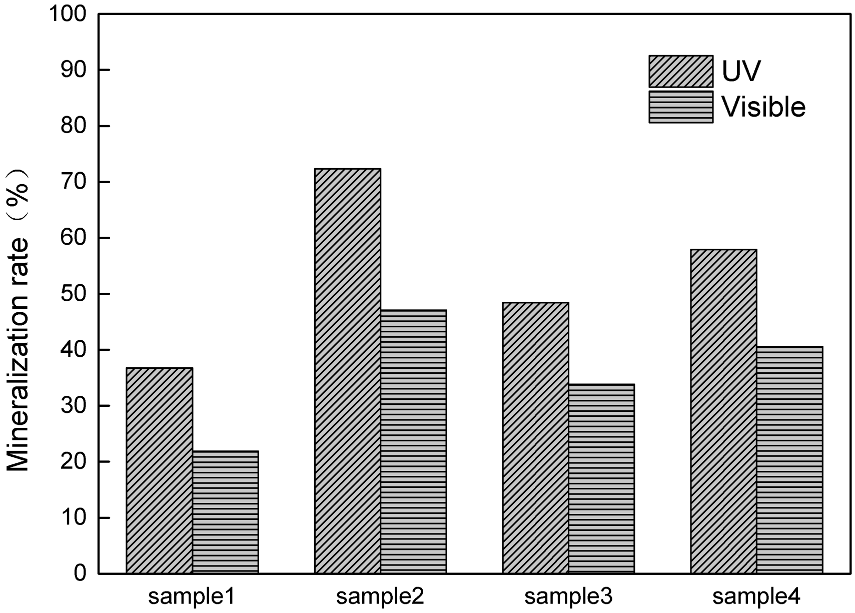

Figure S4. Mineralization rates of MO by the samples under UV and Visible light irradiation. Samples 1–4 denote pure TiO_2 NPs, Ag@TiO_2 NPs-0.5, Ag@TiO_2 NPs-0.25 and Ag@TiO_2 NPs-1.
